# Modulation of Intestinal Flora by Dietary Polysaccharides: A Novel Approach for the Treatment and Prevention of Metabolic Disorders

**DOI:** 10.3390/foods11192961

**Published:** 2022-09-22

**Authors:** Li Zhang, Xinzhou Wang, Xin Zhang

**Affiliations:** 1 Department of Physical Education, China University of Mining and Technology, Beijing 100083, China; 2 Department of Food Science and Engineering, Ningbo University, Ningbo 315211, China

**Keywords:** intestinal flora, dietary polysaccharides, metabolic disorders

## Abstract

Intestinal flora is numerous and diverse, and play a key role in maintaining human health. Dietary polysaccharides are widely present in the daily diet and have a moderating effect on the intestinal flora. Past studies have confirmed that intestinal flora is involved in the metabolic process in the human body, and the change in intestinal flora structure is closely related to the metabolic disorders in the human body. Therefore, regulating intestinal flora through dietary polysaccharides is an effective way to treat and prevent common metabolic diseases and has great research value. However, this area has not received enough attention. In this review, we provide an overview of the modulatory effects of dietary polysaccharides on intestinal flora and the key role of intestinal flora in improving metabolic disorders in humans. In addition, we highlight the therapeutic and preventive effects of intestinal flora modulation through dietary polysaccharides on metabolic disorders, aiming to find new ways to treat metabolic disorders and facilitate future exploration in this field.

## 1. Introduction

Research over the past decades has revealed that the human gut hosts a large and complex array of microorganisms that have co-evolved over thousands of years to form a mutually beneficial and symbiotic relationship with their hosts, an aggregation of microorganisms known as the intestinal flora [1,2]. According to available experimental data, a healthy human intestine hosts more than 100 trillion microorganisms, which is almost the sum of all cells in the body [3,4], and the intestinal flora contains more than three million genes, which is far more than the 23,000 genes that make up the human genome [5]. Nowadays, scientists generally agree that maintaining human health, apart from the body’s genome, much depends on the genome of microorganisms in the human body. Therefore, the intestinal flora is also known as the second genome of the human body, and its importance to human health cannot be overstated [6,7]. In addition, researchers have found that the structural composition of the intestinal flora is dynamic and can be altered by a variety of factors, which have been found in past studies to include age, gender, medication, and dietary habits [8,9,10,11]. Among them, the dietary factor has received great attention from scientists because of its convenience and efficiency. Based on past experiments comparing the effects of a high-fat Western diet and a healthy Mediterranean diet on gut flora, it was found that the diversity of gut flora was significantly lower in Western diet subjects [12], and the content of important gut flora metabolites, such as short-chain fatty acids (SCFAs), was much lower than in Mediterranean diet subjects [13,14,15]. In addition, the prevalence of metabolic diseases caused by the Western diet was much higher than that of the Mediterranean diet [15,16]. We can easily find that the Mediterranean diet has more bioactive substances, such as dietary polysaccharides, in its nutritional composition than the Western diet [15], and we can boldly speculate that the secret of health may lie in these bioactive substances and their close connection with intestinal flora.

Dietary polysaccharides, as one of the typical representatives of bioactive substances, are widely used in our daily diet. Dietary polysaccharides are mainly derived from nature and are found in vegetables, fruits, edible mushrooms, and medicinal plants, which are closely related to daily life [17,18,19]. Many scientists in the past have extensively studied polysaccharides because of their safety, accessibility, and high pharmacological value, and today, their anti-diabetic, anti-tumor, anti-obesity, and other health benefits have been proven [20,21,22,23]. As research continues, scientists have discovered that the beneficial effects of polysaccharides on health appear to be linked to the intestinal flora in our bodies, making research on the effects of polysaccharides on intestinal flora a popular topic again. Nowadays, a large number of experimental results also prove that dietary polysaccharides can indeed have an effect on the composition and structure of intestinal flora and the production of beneficial metabolites of intestinal flora [24,25]. With these experimental data as support, it is also possible to regulate intestinal flora through dietary polysaccharides and have some therapeutic effect on certain diseases, including the most common metabolic diseases, such as type 2 diabetes and hyperlipidemia, which afflict a great number of people in the world [26,27]. This novel and gentle treatment is undoubtedly a boon for these patients and deserves our in-depth attention.

Metabolic diseases, commonly including obesity, type 2 diabetes, and hyperlipidemia, are extremely dangerous to human health [28,29,30]. Although, in the past, scientists have invested a lot of time in studying the causes of these diseases, they have not been able to give a complete answer until now. According to the available experimental results, the factors that trigger the occurrence of metabolic diseases include genetics, dietary habits, lifestyle, and intestinal flora [31,32,33,34]. Among them, the association between intestinal flora and metabolic diseases has made great progress in recent years. In past studies, scientists have found that the structure of the intestinal flora of obese people differs from that of normal people, and subsequent studies have also confirmed that this change in the structure of the intestinal flora causes an increase in calorie production [35,36]. In addition, research has progressed in the treatment of related metabolic diseases by suppressing fecal flora [37,38]. The close association between intestinal flora and metabolic diseases is supported by numerous experimental data and is indisputable. Further, a healthy diet is an important condition to prevent intestinal flora dysbiosis, and we can explore the beneficial effects of a substance in a healthy diet, such as dietary polysaccharides, on intestinal flora to maintain human health and to treat and prevent related metabolic diseases.

Research on the effects of dietary polysaccharides on intestinal flora is ongoing and, based on the data available today, there is reason to believe that regulating intestinal flora through the use of dietary polysaccharides can play a role in the treatment and prevention of metabolic diseases. Although diet permeates people’s daily lives, people often do not care about the specific bioactive substances they can consume from food. In this review, we outline the mechanisms by which dietary polysaccharides regulate intestinal flora and the key role that intestinal flora plays in maintaining normal metabolism in the body. In addition, we highlight the therapeutic and preventive effects of dietary polysaccharides on common metabolic diseases by modulating the composition of the intestinal flora and promoting the production of metabolites from important intestinal flora, and we look into the future development of this field.

## 2. Relationship between Dietary Polysaccharides and Gut Microbiota

### 2.1. Dietary Polysaccharide

Dietary polysaccharides, as the name suggests, are polysaccharides present in our daily diet, including the fruits, vegetables, and herbs we eat daily. Polysaccharides have a relatively complex structure; however, with the development and progress of science and technology, we have gained some understanding of the structure of some polysaccharides and the related sugar units that make up polysaccharides. Broadly speaking, natural polysaccharides are composed of many monosaccharide residues linked by glycosidic bonds [39,40]. Dietary polysaccharides have received a lot of attention in various fields because of their stable, non-toxic, high medicinal value, cheap, and easy-to-obtain advantages [22,29]. Dietary polysaccharides are used in a variety of food applications, such as improving the stability of food emulsions [41,42], or compensating for the lower fat content of low-fat foods, providing an oily texture, and improving the sensory quality of foods [43,44]. Nowadays, they are also used in health care, for example, to improve the immune system or to alleviate a range of metabolic diseases associated with an unhealthy diet, playing an important role in the maintenance of human health [45,46,47]. In summarizing these past studies, it is important to note that the health benefits of dietary polysaccharides are often accompanied by changes in the composition of the intestinal flora and in the levels of important metabolites of the intestinal flora. As the “second genome” of the human body, intestinal flora plays a key role in human health, and the change in its structure will directly affect the normal operation of the human “factory”, which can be an important breakthrough in the research of dietary polysaccharide.

### 2.2. Metabolism of Dietary Polysaccharides by Gut Microbiota

After people take food from the outside world, the food undergoes a series of complex digestive processes in the human body and becomes small molecules of nutrients that can be absorbed and finally used by the body [48]. Among them, some foods rich in polysaccharides can be digested and absorbed by the body to provide energy, such as the starch in rice, but some cannot be digested and absorbed, such as the fiber in vegetables [49,50]. Due to the lack of enzymes in the body, these complex polysaccharides cannot be further digested, but they can be utilized by the intestinal flora and promote the production of related metabolites, which ultimately benefit human health [51,52]. For these dietary polysaccharides that cannot be digested and absorbed by the body, they can be divided into two categories, one is fermentable, and the other is non-fermentable. The non-fermentable polysaccharides will eventually be excreted with feces, while the fermentable polysaccharides will be utilized and degraded by the intestinal flora [27].

To date, a number of intestinal microorganisms have been identified that can utilize dietary polysaccharides, and the mystery lies in the presence of enzymes in these intestinal microorganisms that can degrade these polysaccharides, such as *Bacteroides* and *Firmicutes*, which make up a large percentage of the human intestinal flora [53]. Studies have shown that intestinal flora can produce carbohydrate-active enzymes (CAZymes), and in subsequent studies, CAZymes were found to degrade complex polysaccharides into fermentable monosaccharides, which greatly aids in the utilization and absorption of polysaccharides by the body [54,55]. Therefore, these dietary polysaccharides, which cannot be digested and utilized by the body, become an important source of energy for the intestinal flora, which can provide itself with energy by fermenting these polysaccharides, further promoting the production of metabolites, such as SCFAs [56]. SCFAs are typical of a wide range of products of the intestinal flora, and they consist of a carboxylic acid and a small hydrocarbon chain (carbon chain length of six carbon atoms or less), common ones include propionic acid, acetic acid, and butyric acid [57]. Past studies have shown that SCFAs not only provide energy to intestinal epithelial cells, but also affect other organs in the body through the gut-brain axis and gut-lung axis to ensure their proper functioning [58,59,60]. In addition, SCFAs are also involved in metabolism and play a role in preventing common metabolic diseases, such as type 2 diabetes and hyperlipidemia, which are very important for maintaining human health [61,62]. For instance, mushroom polysaccharides have been shown not to be digested and absorbed by the body itself [63], but can be used by the flora in the human intestine, stimulating the intestinal flora to produce more beneficial metabolites, including the aforementioned SCFAs, thus exerting beneficial effects such as cancer prevention and obesity prevention, and maintaining the health of the body [64,65].

### 2.3. The Regulatory Effect of Dietary Polysaccharides on the Structure of Intestinal Flora

The composition of the intestinal flora is not static and can change under the influence of many factors. Among the many influencing factors, diet is considered to be one of the most likely to shape intestinal flora. Dietary polysaccharides are widely found in vegetables and fruits, are an integral part of our daily diet, and naturally have the potential to reshape our intestinal flora. Some complex structures of dietary polysaccharides, due to the lack of corresponding enzymes in the human body, cannot be effectively digested and used; these dietary polysaccharides will enter the intestinal tract along our digestive tract, giving the intestinal flora opportunities to digest them [25].

**Figure 1:**
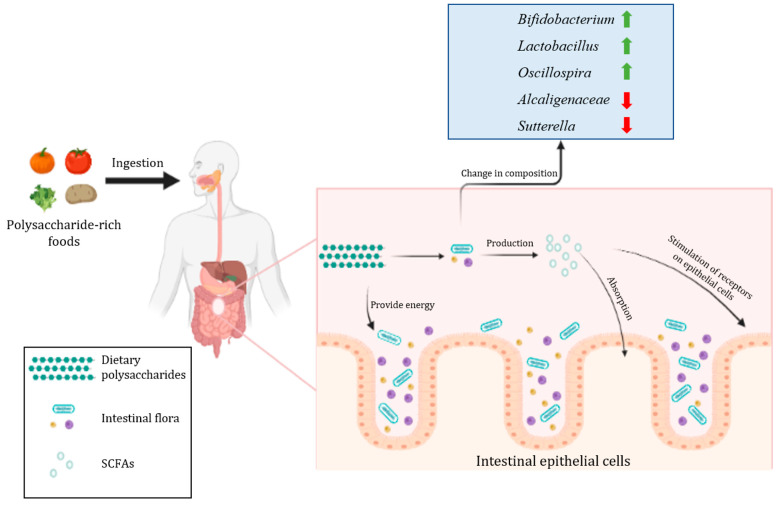
The effect of dietary polysaccharides on intestinal flora.

Dietary polysaccharides are an important source of nutrition for some microorganisms in the intestine that contain enzymes related to the use of dietary polysaccharides, which promote the growth and proliferation of these microorganisms in the intestine, thus altering the composition of the intestinal flora [66]. In addition, dietary polysaccharides also promote the production of important metabolites of the intestinal flora, which changes the growth environment of microorganisms in the intestine and is more favorable to the growth of related microorganisms, further causing changes to the structure of the intestinal flora [66]. Interestingly, according to the study, most of these microorganisms are the ones that are dominant or beneficial in the gut and are an important part of healthy intestinal flora. As an example, in a study of coix polysaccharides, Yin et al. [67] found that the percentage of *Bifidobacterium* and *Lactobacillus* in the intestine increased after the coix polysaccharides intervention compared to the control group, and there was a significant increase in the total number of SCFAs. In another experiment on the regulation of intestinal flora by purple sweet potato polysaccharides, Tang et al. [68] found that the levels of *Bacteroidetes*, *Lachnospiraceae*, and *Oscillospira* increased and the levels of *Alcaligenaceae* and *Sutterella* decreased in the intestine with the intervention of purple sweet potato polysaccharides, and this result confirmed that purple sweet potato polysaccharides have a modulating effect on the structure of intestinal flora. Moreover, there is a large number of experiments that have confirmed the regulatory effect of dietary polysaccharides on the intestinal flora, including common and uncommon foods in our lives [24,69,70]. The effect of dietary polysaccharides on intestinal flora is shown in Figure 1. Dietary polysaccharides act directly on the intestinal flora by acting as a source of energy, increasing the relative abundance of beneficial bacteria in the gut and further promoting the production of important metabolites such as SCFAs, which play a critical role in maintaining health, including the prevention and treatment of metabolic diseases.

## 3. The Role of Gut Microbiota in Metabolic Disorders

### 3.1. Metabolic Disorders and Metabolic Diseases

Everyone has been bothered by illness at some point in their lives. Regarding diabetes, hyperlipidemia, and other common metabolic diseases, the cause of these diseases is mainly the disruption of the body’s metabolism of the relevant substances. For example, diabetes is a metabolic disease caused by disorders of glucose metabolism, which can be subdivided into type 1 diabetes and type 2 diabetes, of which type 2 diabetes is more common [71]. The main reasons for the development of type 2 diabetes can be categorized into two points: inadequate ability of insulin-sensitive tissues to respond to insulin and a defect in the ability of pancreatic beta cells to secrete insulin [28,72]. According to reports, more than 90% of people with diabetes are type 2 diabetics, and in 2017, about 462 million people worldwide suffered from type 2 diabetes, equivalent to 6.28% of the world’s population, and about 1 million people died from type 2 diabetes each year, ranked as the ninth leading cause of death, and, even more frighteningly, according to expert extrapolation, the prevalence of type 2 diabetes will continue to rise, and by 2030, the number of type 2 diabetes patients per 100,000 people is expected to reach 7079 [72,73]. Type 2 diabetes poses a great threat to the health of modern society and needs to receive adequate social attention.

In addition, hyperlipidemia due to abnormal fat metabolism afflicts many people worldwide. Hyperlipidemia occurs when the level of lipids or lipoproteins in the blood is elevated due to abnormalities in the body’s fat metabolism or related functions [74,75]. Hyperlipidemia is one of the factors that induce various types of cardiovascular diseases and can lead to the occurrence of non-ischemic heart failure. According to studies, hyperlipidemia can not only affect the contractile function and electrophysiological response of the heart, but also can cause atherosclerosis to indirectly affect heart function, which makes patients with hyperlipidemia twice as likely to develop cardiovascular diseases as normal people [30,76,77].

Up to now, although we have gained some understanding of the pathogenesis of the above two common metabolic diseases through relentless research, it is not enough to draw a complete conclusion. In summary, the main factors for the development of these diseases include genetics, lack of exercise, and poor dietary habits [78,79,80,81,82]. With the continuous development of the economy and society, the number of cases of metabolic diseases caused by sedentary work style and a high fat and high sugar diet is increasing. A point of interest is that scientists have found a close link between intestinal flora and metabolic diseases, and that the composition and metabolites of intestinal flora will affect the occurrence of metabolic diseases. Given the importance of intestinal flora to human health, this direction has great potential for research.

### 3.2. The Strong Link between Intestinal Flora and Metabolic Disorders

Doctors advise society on how to prevent metabolic diseases, such as increasing exercise appropriately and arranging diet wisely. In the process of “healthy living”, the intestinal flora in our body is also changing, which is closely related to our health. First, based on the available data, we can determine that the structure of the intestinal flora is altered in patients with metabolic disorders compared to healthy individuals. In a study investigating the composition and structure of the intestinal flora of patients with metabolic syndrome, He et al. [83] found that, compared to healthy individuals, patients with metabolic syndrome showed significant dysbiosis of the intestinal flora, as evidenced by an altered abundance of *Firmicutes* and *Proteobacteria*, and a much smaller diversity of intestinal flora than healthy normal individuals. In another experiment, the researchers caused metabolic disorders in mice by giving them a high sugar diet and found that the changes in the intestinal flora of the metabolic disorder mice showed a decrease in the proportion of *Bacteroidetes* and an increase in the proportion of *Proteobacteria* compared to the control group [84]. In addition, there are several experimental data that provide a strong basis for the conclusion that metabolic diseases cause changes in the composition and structure of the intestinal flora, which is the most visual evidence of a strong association between intestinal flora and metabolic diseases [85,86,87].

Secondly, the metabolites associated with intestinal flora are also associated with metabolic disorders. SCFAs, as one of the most important metabolites of the intestinal flora, have been mentioned above, and their role in health has been repeatedly emphasized by scientists. Based on the available experimental data, although we cannot fully determine the exact mechanism by which SCFAs affect human metabolism, there has been some progress in the role played by SCFAs in the metabolic process. Studies have shown that butyrate can provide energy to intestinal epithelial cells and maintain a stable intestinal environment; in addition, SCFAs can act as signaling factors to activate AMP kinase by G-protein coupled receptor 41 (GPR41) and G-protein coupled receptor 43 (GPR43) in cells, including GPR41 and GPR43 in immune cells, liver, and adipose tissue [88,89,90,91]. SCFAs play different roles by binding to GPR41 and GPR43 on different cells, which include the regulation of metabolic disorders. As an example, butyrate can bind to GPR receptors on intestinal endocrine L cells and promote the cellular secretion of peptide YY (PYY) and glucagon-like peptide-1 (GLP-1) [92]. GLP-1 is produced by L cells during feeding and is able to promote insulin secretion in a glucose-dependent state, which is important for lowering blood glucose [61]. PYY is an intestinal peptide that is encoded by the human PYY gene and is released by cells in the human colon and ileum. PYY works by acting on the neuropeptide Y (NPY) receptor to suppress appetite, reduce gastric motility, and increase the absorption of water and electrolytes in the colon [93]. In addition to SCFAs, another metabolite, secondary bile acids, also play an important role. Secondary bile acids are converted from primary bile acids that enter the intestine through an enzyme-catalyzed reaction in the intestinal flora that removes the hydroxyl group [94]. Both primary and secondary bile acids are important functional models in the body, capable of acting on farnesoid X (FXR) and Takeda G-protein-coupled receptor 5 (TGR5) nuclear receptor, whose activation can serve to regulate lipid and glucose endostasis [95,96]. Taking glucose metabolism in the human body as an example, activation of TGR5 by secondary bile acids stimulates the secretion of GLP-1 in intestinal endocrine L cells, the function of which has been explained above. In addition, activation of TGR5 increases muscle energy expenditure and improves sensitivity to insulin, which is of great significance for maintaining normal glucose metabolism in the human body [97,98].

Last but not least, the metabolites of the intestinal flora, in addition to some of the beneficial metabolites mentioned above, there are also metabolites that are harmful to health, and dysbiosis of the intestinal flora will also lead to changes in the levels of these harmful products, posing some risk to the normal metabolism of the body, such as lipopolysaccharide (LPS). LPS is a bacterial surface glycolipid present in the outer membrane of Gram-negative bacteria and is capable of eliciting an immune response associated with inflammation. According to studies, dysbiosis of the intestinal flora will prevent the tight junctions of intestinal epithelial cells leading to increased intestinal permeability and increased production of LPS by dysbiosis of the intestinal flora. These factors ultimately cause more LPS to enter the bloodstream, leading to increased levels of LPS in the blood and its transport to various organs of the body [99,100]. LPS can bind to Toll-like receptor 4 (TLR-4) to transfer into host cells and induce the release of inflammatory factors leading to inflammation [100,101]. It is important to emphasize that LPS can also impede insulin signaling by affecting signaling pathways, causing a decrease in insulin sensitivity in the body and thus jeopardizing normal metabolism [102].

**Figure 2:**
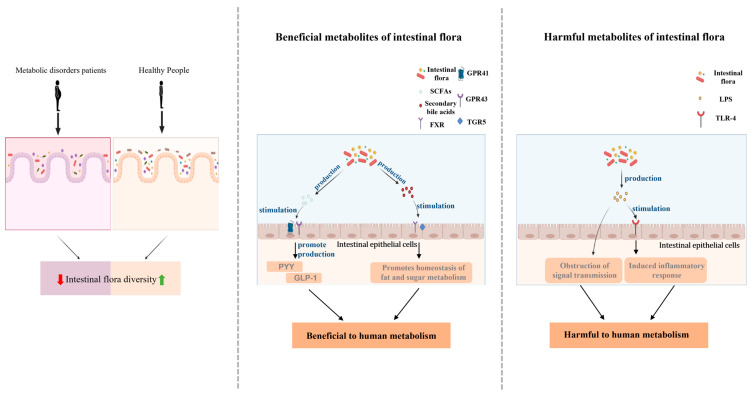
Intestinal flora and metabolic disorders.

In summary, the link between intestinal flora and metabolic disorders is shown in Figure 2. Based on the importance of gut flora for human health, we can speculate that in the future, regulation of gut flora will be a major option for the treatment of metabolic disorders, and there is great potential for research in this area. Exploring the role of intestinal flora in human metabolic processes will help us to reduce the increasing number of patients with metabolic diseases and to solve the health crisis caused by metabolic diseases such as diabetes and hyperlipidemia.

## 4. Dietary Intervention Modulates Gut Microbiota to Prevent and Treat Metabolic Diseases

### 4.1. Treatment and Prevention of Type 2 Diabetes

Type 2 diabetes is a typical representative of metabolic diseases, as described above. As research into type 2 diabetes has advanced, various means of prevention and treatment have emerged; however, by far the most common means remains pharmacotherapy, such as drugs that target β-cells to induce their proliferation or attenuate their apoptosis [103]. While the efficiency of these drugs is to be celebrated, we have to consider the possible side effects of drug therapy and whether patients can afford the expenses required for treatment. It is well known that, excluding genetic factors, the main reason for the increasing number of modern type 2 diabetics is poor dietary habits, and prolonged high-fat and high-sugar diets increase the risk of developing type 2 diabetes [104,105]. Conversely, we can also explore the possibility of dietary interventions for the treatment and prevention of type 2 diabetes from the perspective of diet, in conjunction with gut flora. Of course, this speculation has a certain theoretical basis. Based on the great influence of diet on intestinal flora and the important role intestinal flora plays in the metabolic process of the human body, we have reasons to believe that regulating intestinal flora through diet will become a new option for the prevention and treatment of type 2 diabetes. Dietary polysaccharides in the daily diet are a typical example.

Dietary polysaccharides regulate intestinal flora to prevent and treat type 2 diabetes. In summary, it is mainly the beneficial regulation of the composition and structure of intestinal flora by dietary polysaccharides and the promotion of the production of beneficial metabolites of intestinal flora that ensure the homeostasis of the intestinal environment. Up to now, certain progress has been made in this field of research, which has laid a solid foundation for the wider application of dietary polysaccharides in the prevention and treatment of type 2 diabetes in the future. In a study on the effect of pumpkin polysaccharides in alleviating type 2 diabetes, Liu et al. [106] found that pumpkin polysaccharides had a moderating effect on the intestinal flora of type 2 diabetic rats, increasing the levels of key strains *Bacteroidetes*, *Deltaproteobacteria*, and *Veillonellaceae*, and enhancing the production of SCFAs in rats. The experimental results also showed that pumpkin polysaccharides improved insulin tolerance and decreased serum glucose levels. In addition, dietary polysaccharides can effectively regulate intestinal flora and promote the production of beneficial metabolites of intestinal flora, and also play a certain role in the prevention and treatment of type 2 diabetes, including tea polysaccharides, sea cucumber polysaccharides, and fava bean polysaccharides and other common foods [107,108,109,110,111] (Table 1).

In the past, scientists have linked dietary polysaccharides to the treatment of type 2 diabetes, finding that they can effectively reduce pancreatic β-cell dysfunction and lower blood glucose by enhancing insulin-related signaling pathways, but little research has been conducted into the processes that cause this outcome. With the continuous development of science and technology, scientists are gradually discovering that intestinal flora plays an important role in the connection between the two, which opens a new door for the treatment of type 2 diabetes. However, we should pay attention to the fact that although it is indisputable that dietary polysaccharides can play a therapeutic and preventive role in type 2 diabetes by regulating intestinal flora, in terms of efficiency, dietary polysaccharides are still no substitute for hypoglycemic drugs; although these drugs have certain side effects, such as thiazolidinediones and sulfonylureas [112,113], this also points us to the way forward. Based on the advantages of easy access and high safety of dietary polysaccharides, combined with intestinal flora, a dietary therapy based on dietary polysaccharides may be available in the future to treat type 2 diabetes efficiently, cheaply, and safely.

### 4.2. Treatment and Prevention of Hyperlipidemia

Hyperlipidemia is an excessive level of lipids in the blood due to dysfunction of lipid metabolism, which can cause a variety of diseases, such as coronary heart disease, and is a serious risk to human health [114]. Similar to the treatment of other metabolic diseases, drugs are still the mainstay of treatment for hyperlipidemia today, but from the perspective of social concern, more attention is being paid to the preventive and therapeutic effects of lifestyle changes on hyperlipidemia. As scientists strongly advocate, a healthy lifestyle can indeed reduce the risk of hyperlipidemia, such as an appropriate increase in daily exercise. A study of elderly patients with hyperlipidemia noted that tai chi exercise effectively improved hyperlipidemia and strengthened the cardiovascular system in middle-aged and older adults [82]. In addition, dietary intervention is another tool that can effectively combat hyperlipidemia and is closer to people’s lives than any other method, since life is impossible without diet. From this perspective, scientists advocate a low-sugar, low-fat diet with a balanced nutritional profile [115,116]. However, the understanding of diet for the prevention and treatment of hyperlipidemia is often limited to the stage of “eating this is good for health”, while the nutrients present in food and the role they can play are often ignored. In the context of intestinal flora, diet, as a key factor affecting intestinal flora, can effectively regulate the structure of intestinal flora and metabolites to prevent and treat hyperlipidemia, and this area needs to be given sufficient attention [117,118].

Studies have shown that a variety of natural bioactive ingredients in foods can play a role in regulating intestinal flora and ultimately improving hyperlipidemia, of which dietary polysaccharides, as a typical representative, are once again a popular research topic [45,119,120]. By summarizing several animal experiments, we found that dietary polysaccharides effectively regulated the disordered intestinal flora of hyperlipidemic rats, as shown by reducing the ratio between Firmicutes and Bacteroidetes, and increasing the relative abundance of probiotic bacteria. Among them, what caught the scientists’ attention was that the intervention of dietary polysaccharides upregulated the abundance of SCFAs-producing bacteria such as *Bacteroides* and *Paraprevotella*, which directly led to an increase in the level of SCFAs in the organism, and the final experimental results also showed a significant decrease in the blood lipid content of the experimental animals, with dietary polysaccharides playing a role in regulating intestinal flora, lowering blood lipids and improving hyperlipidemia [120,121,122]. These data provide strong support that dietary polysaccharides can be a new option for the prevention and treatment of hyperlipidemia. However, while we have only been able to collect very little data from human experiments, the in vitro experiments on human intestinal flora and dietary polysaccharides have also given us great insight. An in vitro structure of an experiment by Gao et al. [123] on the effect of kelp polysaccharides on lipid metabolism in human intestinal flora showed that kelp polysaccharides upregulated the abundance of *Firmicutes* and promoted the production of SCFAs. In addition, many other in vitro experiments simulating digestion and fermentation in the gastrointestinal tract have yielded similar results, which add credibility to the data from such experiments. It is not difficult to find that the effect of dietary polysaccharides on human intestinal flora is similar to the animal experiments mentioned above, which indirectly verifies that dietary polysaccharides can promote human lipid metabolism and ultimately improve hyperlipidemia by regulating intestinal flora even in humans [123,124].

Based on the advantages of dietary polysaccharides and the importance of intestinal flora, we have good reasons to believe that, in the future, dietary polysaccharides could become a new option for the treatment of hyperlipidemia patients in addition to drug therapy, and also increase the prevention of hyperlipidemia at the social level. Recently, fecal intestinal flora transplantation has become an effective treatment [125], we can boldly speculate that we can use bioactive substances such as dietary polysaccharides to purposefully regulate the composition of intestinal flora in vitro and transplant them into patients to play a beneficial role in a safe manner. The treatment of intestinal flora by polysaccharides has undoubtedly brought a boon to patients with hyperlipidemia, who can receive safe and effective treatment without having to bear a lot of medical expenses. Of course, how to improve the therapeutic effect of this method and how to apply it in practice is still the focus of our future research.

## 5. Conclusions

As a common bioactive substance in food, dietary polysaccharides can effectively regulate the composition structure of intestinal flora and promote the production of beneficial metabolites of intestinal flora. Intestinal flora and its metabolites are important participants in the human body’s metabolic process, and intestinal flora’s homeostasis can effectively prevent the emergence of metabolic disorders, which can play an effective role in the treatment and prevention of type 2 diabetes, hyperlipidemia, and other metabolic diseases. Although the method of regulating intestinal flora through dietary polysaccharides has certain advantages in many aspects compared with traditional drug therapy, it needs further investigation whether it can replace traditional treatment methods or achieve the same therapeutic effect.

## Figures and Tables

**Table 1:** Dietary polysaccharides regulate intestinal flora in the treatment of type 2 diabetes.

Polysaccharide Sources	Experimental Model	Affected Intestinal Flora	Experimental Results	Reference
Pumpkin	Rats with type 2 diabetes mellitus	The relative abundance of *Bacteroidetes*, *Deltaproteobacteri*, and *Veillonellaceae* increased	Diabetes symptoms have subsided	[106]
Tea		The relative abundance of *Lachnospira*, *Victivallis*, *Roseburia*, and *Fluviicola* was recovered		[107]
Sea cucumber		The relative abundance of beneficial bacteria increased and that of harmful bacteria decreased		[108]
*Plantago asiatica*		The relative abundance of *Bacteroides vulgatus, Prevotella loescheii*, and *Bacteroides vulgates* increased		[110]
*Ganoderma lucidum*		The relative abundance of harmful bacteria *Aerococcus*, *Ruminococcus* decreased and the beneficial bacteria *Parabacteroides* and *Bacteroides* increased		[111]

## Data Availability

No new data were created or analyzed in this study. Data sharing is not applicable to this article.
